# Phagocytic Function Analyses of GAB_B_R-Related Microglia in Immature Developing Epileptic Brain Based on 10× Single-Nucleus RNA Sequencing Technology

**DOI:** 10.3390/biomedicines13020269

**Published:** 2025-01-22

**Authors:** Yunhao Gan, Xiaoyue Yang, Tianyi Li, Ziyao Han, Li Cheng, Lingling Xie, Li Jiang

**Affiliations:** 1 Department of Neurology, Children’s Hospital of Chongqing Medical University, National Clinical Research Center for Child Health and Disorders, Ministry of Education Key Laboratory of Child Development and Disorders, Chongqing 400014, China; yunhaogan28@163.com (Y.G.); yangxiaoyue97@163.com (X.Y.); litianyihka@126.com (T.L.); ziyaohan0410@163.com (Z.H.); 480867@hospital.cqmu.edu.cn (L.C.); 2 Chongqing Key Laboratory of Child Neurodevelopment and Cognitive Disorders, Chongqing Medical University, Chongqing 400014, China

**Keywords:** epilepsy, GAB_B_R-associated microglia, GAB_B_R2, microglia, phagocytic pruning

## Abstract

**Background**: Epilepsy is a neurological disorder defined by the occurrence of epileptic seizures, which can significantly affect children, often leading to learning and cognitive impairments. Microglia, the resident immune cells of the central nervous system, are essential in clearing damaged neurons through phagocytosis. Notably, GAB_B_R-associated microglia have been implicated in regulating phagocytic activity. Since the phagocytic function of microglia is critical in the pathogenesis of epilepsy, this study aims to investigate the role of GAB_B_R-associated microglia in the development of the immature brain following epileptic seizures. **Methods:** Epilepsy was induced in a mouse model by the intraperitoneal injection of KA. Changes in the expression of the GAB_B_R-related gene, GAB_B_R2, in hippocampal microglia were analyzed using single-nucleus RNA sequencing (snRNA-seq). Cognitive and emotional changes in the mice were assessed through behavioral analyses. The expression of GAB_B_R2 was semi-quantitatively measured using Western blotting, quantitative reverse transcription PCR, and immunofluorescence. Additionally, the spatial relationship between GAB_B_R2 and hippocampal neurons was evaluated using Imaris software. **Results:** The snRNA-seq analysis revealed that GAB_B_R2 expression was elevated in activated microglia in the hippocampus during chronic epilepsy compared to the early phase of seizures. Behavioral assessments demonstrated heightened anxiety levels and learning and memory impairments in the chronic epilepsy group compared to the control group. GAB_B_R2 expression was upregulated in chronic epilepsy. Three-dimensional reconstruction analyses revealed a significantly increased contact volume between GAB_B_R-associated microglia and neurons in the chronic epilepsy group compared to the control group. **Conclusions**: GAB_B_R-associated microglia significantly contribute to the progression of immature brain diseases by promoting neuronal phagocytic activity.

## 1. Introduction

In recent years, the prevalence of epilepsy among children and adolescents has steadily increased. Abnormal and excessively synchronized neuronal activity in the brain often results in poor prognoses and elevated mortality rates [1,2,3]. Although various antiepileptic drugs (AEDs) can mitigate and control seizures, these treatments are frequently associated with significant side effects and resistance, posing considerable safety concerns [4,5,6]. Epilepsy is a chronic neurological disorder characterized by recurrent, transient brain dysfunction caused by abnormal neuronal discharges, leading to repeated seizures.

In infants and young children, seizures represent one of the most common neurological emergencies, with approximately 5% of children experiencing such episodes [5,7]. Status epilepticus (SE), a severe and prolonged seizure, affects 17–23 per 100,000 children and carries a mortality rate of 16%. Current treatments primarily rely on AEDs and acute antispasmodic medications [7]. However, despite the availability of various AEDs, approximately 20% of pediatric epilepsy cases remain difficult to manage. Therefore, the development of novel therapeutic strategies to treat pediatric epilepsy and protect the developing brain is imperative.

Extensive research has primarily focused on the abnormal neurophysiological processes of epileptic neurons, yet the phagocytic activity of microglia in clearing synapses and dead neurons is equally critical in epilepsy pathogenesis [8,9,10]. Investigating the role of microglial phagocytosis may offer valuable insights into the mechanisms underlying epilepsy. Previous studies have demonstrated that knocking out TREM2 in epilepsy models reduces microglial proliferation and impairs their ability to clear damaged hippocampal neurons [11]. This reduction in the selective phagocytosis of inhibitory synapses by microglia may disrupt the balance of excitatory and inhibitory (E/I) neurotransmission, thereby contributing to seizure development and symptom severity [12]. Despite these findings, the regulatory mechanisms governing microglial phagocytosis in the brain remain poorly understood.

Gamma-aminobutyric acid (GABA) serves as the primary inhibitory neurotransmitter in the central nervous system, mediating inhibitory synaptic signaling through interactions with GAB_B_ receptors (GAB_B_R) [13,14]. As a G protein-coupled receptor, GAB_B_R induces neuronal hyperpolarization and reduces presynaptic neurotransmitter release by regulating potassium (K⁺) levels and inhibiting calcium (Ca^2^⁺) release [2,15]. Research has demonstrated that microglia express a significant amount of GAB_B_R, which plays a critical role in modulating immune responses in the brain through GABA signaling [16,17,18]. Despite these findings, the specific role of GAB_B_R-associated microglia in the development of epilepsy in the immature brain remains poorly understood.

We established an epilepsy model through intraperitoneal injection of kainic acid (KA), which induces SE and leads to spontaneous recurrent seizures (SRSs) during the chronic phase of epilepsy [19]. Our findings revealed that KA-induced epileptic mice exhibited significant cognitive impairments in the chronic epilepsy stage. Furthermore, microglia associated with GAB_B_R demonstrated an enhanced capacity to phagocytose damaged hippocampal neurons. Collectively, these results suggest that GAB_B_R-associated microglia may play a pivotal role in neuronal phagocytosis in developmental epilepsy and represent a potential therapeutic target for early intervention and the treatment of epilepsy.

## 2. Materials and Methods

### 2.1. Animals

In this study, fourteen-day-old male C57BL/6 mice, sourced from the Laboratory Animal Center of Chongqing Medical University, Chongqing, China, were housed collectively in our SPF facility and subjected to a 12 h light/dark cycle, with ad libitum access to food and water. In this study, no more than five mice were housed per cage. Ethical approval for all procedures was granted by the Commission of Chongqing Medical University for Ethics of Experiments on Animals, and all were conducted in compliance with international standards. All procedures for this study were approved by the Animal Care and Committee of Chongqing Medical University (Approval number: IACUC-CQMU-2024-0639). The experimental design was both randomized and double-blind, involving immature developing mice aged P14. Efforts were made to minimize animal suffering and to reduce the number of animals used throughout the experiments.

### 2.2. Kainic Acid (KA) Model of Epilepsy

The intraperitoneal (i.p.) administration of KA to induce status epilepticus (SE) was established using a previously described method [20]. Ninety-six mice were administered a single dose of KA (0.75 µL/g, Sigma–Aldrich, St. Louis, MO, USA), resulting in continuous SE for 30 min, followed by the administration of 1 mg/kg (i.p.) diazepam to terminate the condition. Seizure severity was assessed using Racine’s scale: stage 0, no response or behavior arrest; stage 1, chewing or facial twitches; stage 2, chewing and head nodding or wet dog shakes; stage 3, unilateral forelimb clonus; stage 4, bilateral forelimb clonus and rearing; stage 5, bilateral forelimb clonus, rearing, and falling [21].

In this investigation, the majority of mice exhibited SE at stages 3–5, with an approximate mortality rate of 20%. The control group, comprising eighty-seven mice, received solely 0.1% phosphate-buffered saline (PBS) and diazepam. The experimental groups were designated as the 14-1-SC group, 14-7-SC group, 14-14-SC group, and 14-28-SC group. Correspondingly, the control groups were named the 14-1-Control group, 14-7-Control group, 14-14-Control group, and 14-28-Control group, respectively.

### 2.3. Reagents and Antibodies

The following antibodies were used: IBA1 and NeuN were from Novus and Pronteintech, respectively (Cat# NB100-1028, Littleton, CO, USA, RRID: AB_3148646; Cat# 66836-1-Ig, Wuhan, China, RRID: AB_2880708). Antibodies against GAB_B_R2 for immunofluorescence (IF) and Western blot (WB) analysis came from Santacruz (Cat# sc-393286, CA, USA, RRID: AB_3668753). Antibodies against β-actin for WB came from Proteintech (Cat# HRP-60008, Proteintech, Wuhan, China, RRID: AB_2722565). Donkey anti-rabbit, Donkey anti-mouse and Donkey anti-goat secondary antibodies and hydrochloric acid (DAPI) were purchased from Thermo Fisher (A10042, A-31571, A-11055, 62247, Waltham, MA, USA).

### 2.4. Immunostaining, Imaging, and Quantification

This study utilizes frozen sections for immunofluorescence staining, as previously outlined in reference [22]. The slices were washed three times with PBS, permeated, and blocked using a solution containing 5% BSA and 0.1% Triton X-100 (CAS 9036-19-5; Sigma-Aldrich, St. Louis, MO, USA) at room temperature for one hour. Subsequently, the tissue slices were incubated with the primary antibody in a blocking solution overnight at 4 °C. Following multiple washes, the blocking solution was augmented with the secondary antibody containing DAPI and incubated for 1 h. Images were acquired using an Olympus microscope (VS200, Spin SR, Tokyo, Japan). To determine the cellular localization of GAB_B_R2, 3 random images were captured from different fields within the same region using a 60× oil immersion microscope. The staining intensity of GAB_B_R2-positive microglia was quantified using Image J. Each brain cell was delineated as an individual region of interest (ROI), and the mean pixel density was calculated [23].

### 2.5. Three-Dimension (3D) Reconstruction and Quantification

The quantitative phagocytosis method was described previously [23]. For 3D reconstruction, 20 μm confocal stacks were analyzed with Imaris software (version 9.0.1, Bitplane). High-resolution confocal images were subjected to 3D reconstruction to visualize GABBR2-positive microglia, employing co-staining with IBA1, GABBR2, and NeuN. Subsequently, the reconstructed surface volume of IBA1-engulfing NeuN was recorded and analyzed.

### 2.6. Single-Nucleus RNA-Seq

The original Fastq files for the 14-1-Control, 14-1-SC, 14-28-Control, and 14-28-SC mouse hippocampus datasets were generated using 10× Genomics technology for library preparation, quality control, and sequencing. The raw data files were processed with Cell Ranger v7.1.0 software for subsequent analysis. Seurat (version 4.0.5) was employed to load the processed data for each dataset into R individually. Cell quality was assessed based on the following criteria: (1) the total UMI count (library size) per cell was less than 30,000; (2) the number of detected genes ranged between 200 and 7000; (3) the percentage of mitochondrial gene expression was below 5%; and (4) cells with more than 200 detected genes were selected for further analysis. Doublets were removed using the default settings of DoubletFinder for each sample. The number of primitive cells in the 14-1-Control, 14-1-SC, 14-28-Control, and 14-28-SC mouse hippocampal datasets were 12,488, 12,924, 12,392, and 14,911, respectively. After filtering the cells based on the above parameters, the cell numbers were 10,839, 11,011, 11,718, and 11,177, respectively.

After filtering out low-quality cells, the Integration method in R was used to process and integrate the data with default parameters. Integration involved comparing cells across different batches or experimental conditions based on the “origin.dent” variable. Following the integration of selected anchor points, dimensionality reduction and clustering were performed on the combined dataset to identify cell subpopulations. Cell clusters in each sample were identified by analyzing the top 20 principal components (PCs) of highly variable genes (HVGs).

Seurat’s FindAllMarkers function was then employed to identify marker genes for each cell cluster, which were subsequently annotated manually. To classify the major cell lineages, a set of established marker genes from previous studies was utilized. For astrocytes, markers included Glul, Slc1a3, and Aqp4; for microglia, Cx3cr1, Hexb, and P2ry12; for neurons, Map2, Syn3, and Rbfox3; for oligodendrocytes, Mog, Mag, and Mbp; and for endothelial cells, Fn1, Hmcn1, and Cped1.

After identifying the main cell lineages, the Subset function was used to further perform dimensionality reduction and clustering specifically on microglia cells to identify their subpopulations. For resting microglia, marker genes included Cx3cr1, P2ry12, and Tmem119, while for activated microglia, markers such as Apoe, Cd40, and Arg1 were used. In this phase of the experiment, data visualization was conducted using the DimPlot, DotPlot, EigenPlot, and VlnPlot functions, while cell proportions were calculated and displayed using ggplot. Additionally, manual adjustments were applied during this stage to prevent excessive clustering.

### 2.7. Open Field Test (OTF)

An OFT was conducted in an arena measuring 40 cm × 40 cm × 40 cm. The specific experimental method is as described earlier [24]. In this test, each mouse was positioned at the center of the arena and permitted to explore for a duration of 10 min. Recorded metrics included the average speed and the duration of time spent in the central area. The arena was cleaned with 70% ethanol between mice. The percentage of time spent in the central area was subsequently compared between the two groups. A reduced duration in the central area was indicative of anxiety and depression-like behaviors in the experimental mice.

### 2.8. Elevated Plus Maze (EPM)

The EPM apparatus comprises four rectangular arms, each measuring 50 cm × 10 cm, connected by a central square platform with dimensions of 10 cm × 10 cm. The apparatus includes two closed arms and two open arms, with the closed arms being enclosed by opaque walls that are 40 cm in height on three sides. During the experimental procedure, each subject was placed at the central platform and allowed to explore the maze freely for a duration of 5 min. Following the assessment of each subject, the EPM was disinfected using 75% ethanol. Subsequently, the trajectory length, velocity, and duration spent in both the open and closed arms were quantified and analyzed.

### 2.9. Novel Object Recognition (NOR)

As previously described, an NOR test was conducted in an open field measuring 40 cm × 40 cm × 40 cm [25]. The animals underwent a 5 min training period, during which they were placed in the center of the arena containing two identical objects. Exploratory behavior, defined as the time spent investigating each object, was recorded. After a 24 h interval, the subjects were reintroduced into the arena for the testing phase, during which one of the original objects was replaced with a novel object. The time spent exploring both the familiar and novel objects was measured once again. The results are expressed as the percentage of time spent exploring each object during training or testing.

### 2.10. Y Maze

All mice were subjected to a Y maze test, as previously described, to assess working memory impairment [26]. The Y maze apparatus consists of three arms, each measuring 30 cm × 6 cm × 15 cm. During the test, a mouse is placed in one arm and permitted to explore all three arms freely for a duration of 8 min. The spontaneous alternation rate is subsequently calculated using software. In the context of the Y-maze test, a reduced alternation rate is indicative of impaired working memory.

### 2.11. Morris Water Maze (MWM)

An MWM test was conducted, as previously described, in a circular pool with a diameter of 110 cm and a water temperature of 24 °C [27]. The platform, measuring 10 cm in diameter, is submerged 1 cm below the water surface. The test involves placing each mouse at four quadrants’ starting positions daily, providing 60 s to locate the platform during a 5-day training experiment. Should the mouse fail to reach the platform within this period, it is then guided to the platform. Once the mouse locates the platform, it is maintained on the platform for 15 s. The escape latency is recorded using software. During the testing day, the platform is removed, and the mice are allowed to swim freely in the pool for 60 s. Relevant memory abilities are quantified using software.

### 2.12. Western Blot (WB)

The immunoblotting procedure was performed as previously described [28]. Briefly, total protein was extracted using RIPA buffer (RIPA–phosphatase inhibitor cocktail = 100:1) with the addition of 5× loading buffer. Equal amounts of protein were separated on a 10% SDS-polyacrylamide gel and transferred onto a nitrocellulose membrane (GVHP29325, Millipore, Billerica, MA, USA). The membrane was incubated with primary antibodies overnight at 4 °C. The next day, the membrane was incubated with appropriately diluted secondary antibodies for 1 h at room temperature. Protein bands were visualized using a chemiluminescence WB detection reagent (BJ0004A, Biosharp, Hefei, China). The unprocessed image, without WB cropping, is provided in Supplemental Figure S1.

### 2.13. qRT–PCR (Quantitative Reverse Transcription-Polymerase Chain Reaction)

Total RNA from cultured and tissues was extracted using the FastPure Cell/Tissue Total RNA Isolation Kit (Vazyme Nanjing, China) and reverse-transcribed using the ABScript III RT Master Mix qPCR with gDNA Remover (ABclonal, Wuhan, China) following the manufacturer’s instructions. Real-time PCR was conducted on a Biorad 96 Touch machine (Bio-Rad, Hercules, CA, USA) using the 2× Universal SYBR Green Fast qPCR Mix (ABclonal, Wuhan, China). β-actin served as the internal control. The cycle threshold (Ct) values of the target gene transcripts were normalized to the average Ct of the housekeeping gene β-actin in each reaction. Relative transcript levels were quantified using the comparative Ct (^∆∆^Ct) method. Primer sequences for the qRT-PCR analysis are provided below:
GAB_B_R2: Forward: 5′-AGACTCCACAGCAATACGAAAGA-3′
Reverse: 3′-TTCATGGCGTTGAGGATGATTCT-5′β-actin: Forward: 5′-AGTGTGACGTTGACATCCGTA-3′
Reverse: 3′-GCCAGAGCAGTAATCTCCTTC-5′

### 2.14. Statistics and Reproducibility

According to previous research [29], the sample size for this experiment was calculated with the formula E = total number of animals in each group, ensuring each experimental design an E value exceeding 10. Following established research guidelines, the significance threshold was established at α = 0.05 and the power at 0.80. Consequently, the Wilcoxon Mann–Whitney test determined the minimum effective sample size between the two groups to be N = 3 [30]. This study acknowledges several limitations, including the lack of an initial sample size calculation. However, due to the inherent variability and uncontrollability of the research, a sample size of n = 5 mice was chosen. The most recent iteration of GraphPad Prism 9 (GraphPad Software) was employed for statistical analyses and graphical representations. The Shapiro–Wilk test was utilized to assess the normality of the data distribution. Depending on the results of the normality test, the statistical significance of differences between the two groups was evaluated using either the unpaired two-tailed Student’s *t*-test or the Mann–Whitney U test. *p* > 0.05 was considered indicative of statistical significance. All values are presented as the mean  ±  standard deviation (SD). Non-parametric data are presented as median ± interquartile range.

## 3. Results

### 3.1. Integration and Cell Type Identification of Single Nuclear RNA Sequencing Data from Normal and Epilepsy Groups in Immature Developing Brain

**Figure 1:**
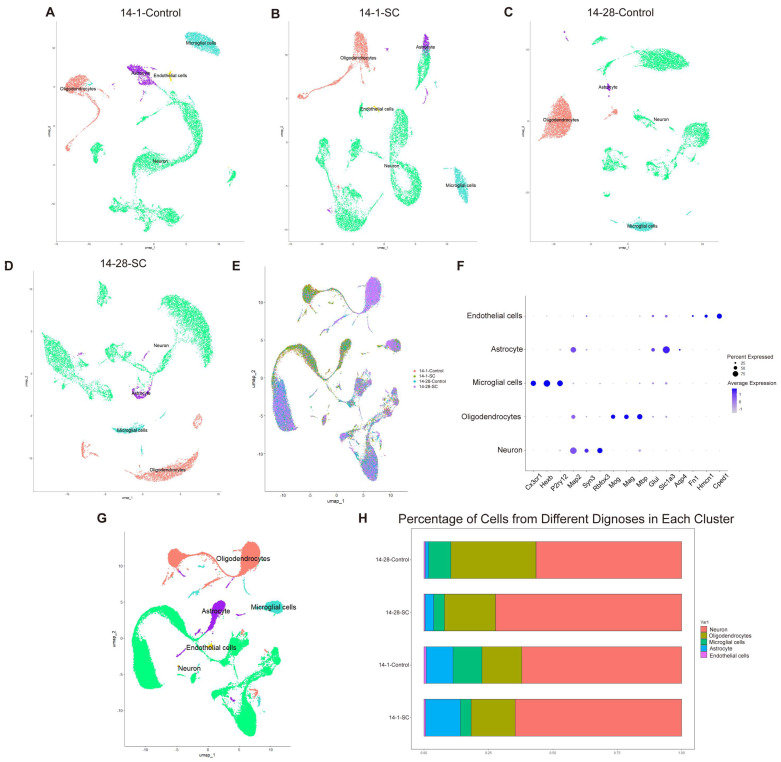
**snRNA-seq analysis in developmental mice after seizures.** (**A**) UMAP visualization of the 14-1 Control group. (**B**) UMAP of 14-1-SC group. (**C**) UMAP of 14-28-Control group. (**D**) UMAP of 14-28-SC group. (**E**) UMAP distribution of cells, annotated by cohort. (**F**) Dot plot showing selected differentially expressed genes for each cluster. (**G**) UMAP distribution of cells, colored by major cell types. (**H**) Histogram illustrating the percentage of cells from different cell types across the four groups.

In this study, we initially performed cell quality control and data dimensionality reduction on datasets from both the normal and epilepsy groups. Subsequently, we conducted UMAP analysis on each dataset to define cell populations across four datasets (Figure 1A–D). Next, we integrated four sets of snRNA-seq data, applying rigorous cell quality control and data dimensionality reduction. This integration significantly enhanced the diversity and scale of the data, providing a robust foundation for subsequent analyses. This integration significantly enhanced the diversity and scale of the data, providing a robust foundation for subsequent analyses (Figure 1E).

Using the “FindAllMarkers” function in Seurat, we classified the cells into four primary types based on established marker genes [23]. These cell types include microglia, marked by *Cx3cr1*, *Hexb*, and *P2ry12*; neurons, marked by *SYN3*, *Map2*, and *RBFOX3*; myelin-forming glial cells, marked by *Mog*, *Mag*, *Mbp*, and *PLP1*; astrocytes, marked by *Glul*, *Slc1a3*, *Aqp4*, and *GAD2*; and endothelial cells, marked by *Fn1*, *Hmcn1*, and *Cped5*. As shown in Figure 1F-G, the distinct marker genes clearly delineate different clusters, and cells from all groups are evenly distributed across these clusters. These results confirm the successful integration of the four datasets and the accurate annotation of the corresponding cell types.

By comparing the distribution of cell types across different populations, we identified cell types that were differentially distributed in specific populations. The analysis of cell populations revealed significant differences between the normal immature brain group and disease groups. In all four immature brain groups, neurons were the predominant cell type, followed by a smaller proportion of dendritic cells. The distribution of cell types, including neurons, oligodendrocytes, microglia, astrocytes, and endothelial cells, varied across the 14-1-Control, 14-1-SC, 14-28-Control, and 14-28-SC groups (Figure 1H). Specifically, neurons constituted 62%, 64%, 57%, and 72% of cells, respectively; oligodendrocytes constituted 16%, 17%, 33%, and 20%; microglia constituted 11%, 4%, 8%, and 4%; astrocytes constituted 10%, 14%, 1%, and 3%; and endothelial cells constituted 0.8%, 0.5%, 0.5% and 0.4%.

### 3.2. The Pressure Profiles of Normal and Epileptic Subgroups of Microglia in the Immature Developing Brain Reveal the Functional Characteristics of GAB_B_R-Relative Microglia

**Figure 2:**
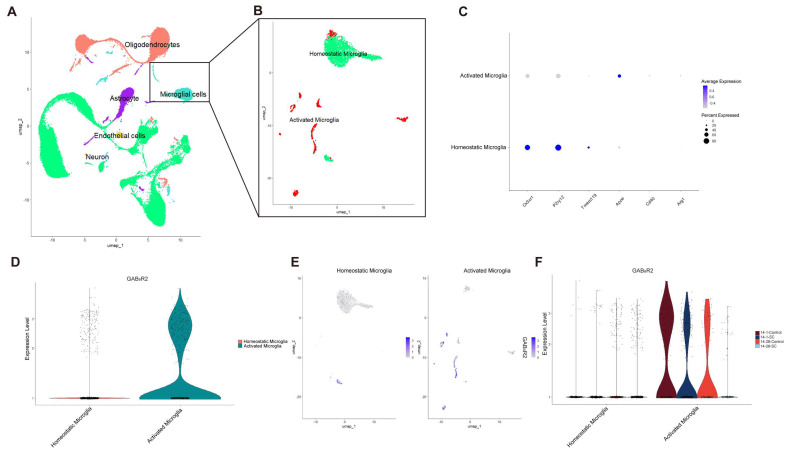
**snRNA-seq analysis of GAB_B_R-relative microglia in developmental mice.** (**A**,**B**) The UMAP plot illustrates the reclustering of microglia into distinct subpopulations. (**C**) The dot plot highlights differentially expressed genes across clusters, with dot size indicating the proportion of nuclei expressing the gene in each cluster and color representing the mean expression level. (**D**) The violin plot visualizes the distribution of GAB_B_R2 specific transcriptional feature scores across microglial subpopulations. (**E**) The UMAP plot depicts the spatial distribution of GAB_B_R2 specific transcript expression, with the highest expression observed in activated microglia. (**F**) Another violin plot presents the distribution of GAB_B_R2 specific transcriptional features across various microglial subgroups.

Microglia, as resident macrophages in the brain, play essential roles in monitoring the brain environment, phagocytosing dead or damaged neurons, and remodeling synapses during the pathological progression of epilepsy [31]. Notably, microglia are crucial in the onset and progression of immature brain diseases associated with epilepsy [32,33]. To investigate their function in both normal and epileptic immature brains, we further subdivided the microglial population into two subgroups, each characterized by distinct marker genes and transcription factors (Figure 2A–C). For instance, steady-state microglial subpopulations predominantly express Cx3cr1, P2ry12, and Tmem119, which are linked to immune surveillance [34,35,36]. Conversely, activated microglial subtypes primarily express Apoe, Cd40, and Arg1, which are associated with neuroinflammatory responses and have been implicated in disease pathology. These subtypes also contribute to synaptic pruning and the clearance of aging neurons and extracellular debris in epilepsy models [36,37,38].

Next, we compared the expression of GAB_B_R2 across different microglial subgroups. Interestingly, GAB_B_R2 was predominantly expressed in activated microglia (Figure 2D,E), suggesting that its function in immature brains is closely linked to the activity of these cells. To further elucidate the expression trajectory of GAB_B_R-associated microglia, we compared the expression levels of resting and activated microglia across four groups. Using the AddModulus Core method to calculate the GAB_B_R2 expression score, we observed significant differences in activated microglia between the 14-1-Control and 14-1-SC groups compared to the 14-28-Control and 14-28-SC groups (Figure 2F). Notably, GAB_B_R2 expression was predominantly enriched in the 14-28-SC group, highlighting its potential regulatory role in the brains of chronic epilepsy models.

### 3.3. Decline in Cognitive and Learning Function in Chronic Epilepsy Mice

Emotional and cognitive disorders are commonly reported in epilepsy patients and corresponding mouse models [39,40,41,42]. This result focused on the cognitive and learning functions in epileptic mice during the chronic epilepsy phase post KA administration.

**Figure 3:**
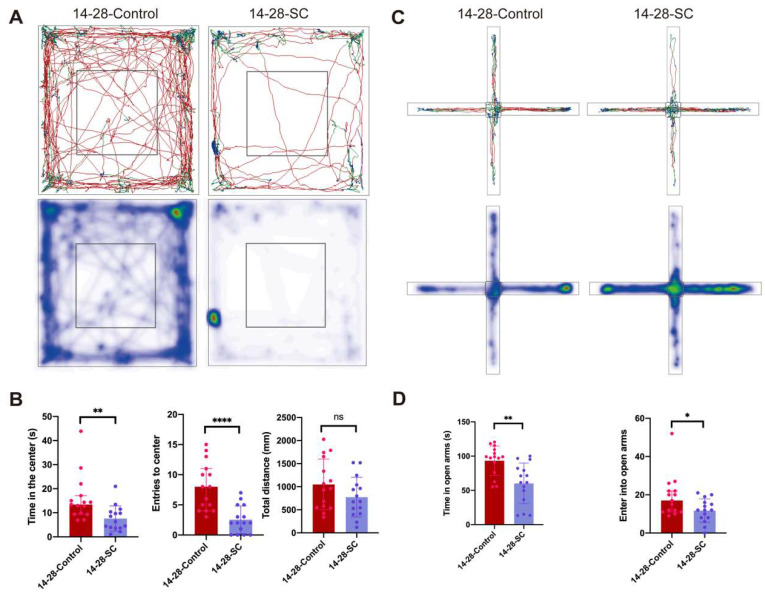
**Detection of emotional changes of mice after intraperitoneal KA.** (**A**) Representative track and heat maps of a 14-28-Control and a 14-28-SC mouse in the OFT. (**B**) 14-28-SC mouse spent significantly less time and entries in the center of OFT as compared to 14-28-Control group, but the total distance had no significant difference. (**C**) Representative track and heat maps of a 14-28-Control and a 14-28-SC mouse in the EPM. (**D**) Compared with the 14-28 control group, the test time and number of entries of the 14-28-SC group in the EPM open arm were significantly reduced. When compared with 14-28-Control group, ^ns^ *p* > 0.05, * *p* < 0.05, ** *p* < 0.01, and **** *p* < 0.0001; data are reported as the mean  ±  SD; unpaired two-tailed Student’s *t*-test.

OFT was utilized to assess the spontaneous activity and exploratory behavior of mice. Results indicated that a notable decrease in both the number and duration of entries into the central area was observed, suggesting heightened anxiety in the unfamiliar environment (Figure 3A,B). In addition, compared to the control group, the total moving distance of epilepsy mice had no difference, indicating that there was no difference in motor ability between the two groups. (Figure 3B). EPM is a widely used behavioral test in research to assess anxiety-like behavior in mice. The findings revealed that compared to the control group, both the frequency and duration of entries into the open arms by chronic epilepsy mice were significantly reduced, indicating severe anxiety (Figure 3C,D).

**Figure 4:**
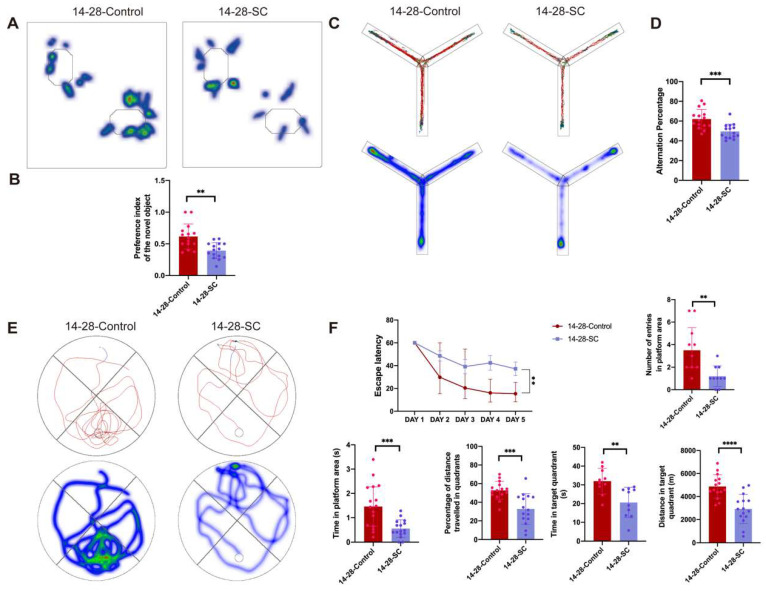
**Detection of cognitive and learning function changes in mice after intraperitoneal KA.** (**A**) Representative heat maps of a 14-28-Control and a 14-28-SC mouse in the NOR. (**B**) Compared to the 14-28-Control group, a decrease in the preference index for new objects in 14-28-SC group. (**C**) Representative track and heat chart of Y maze test. (**D**) Quantification of the alternation rate. (**E**) Representative track and heat maps of a 14-28-Control and a 14-28-SC mouse in the MWM. (**F**) Quantification of the escape latency, number of entries in the platform area, time in the platform area, percentage of distance traveled in quadrants, time in target quadrant and distance in the target quadrant. When compared with the 14-28-Control group, ** *p* < 0.01, *** *p* < 0.001 and **** *p* < 0.0001; data are reported as the mean  ±  SD; unpaired two-tailed Student’s *t*-test.

The NOR experiment was employed to assess learning and memory capabilities. Results indicated a decrease in the preference index for new objects in the 14-28-SC group compared to the 14-28-Control group (Figure 4A,B). Furthermore, the Y-maze test revealed a diminished alternation rate, suggesting impaired working memory in the epileptic mice. Additionally, a lower spontaneous alternation rate was observed in the 14-28-SC group compared to the 14-28-Control group (Figure 4C,D).

Subsequently, the MWM test was conducted to evaluate the learning ability and spatial memory of chronic epileptic mice. During the training phase (over 5 consecutive days), epileptic mice spent more time searching for the platform than the control group, indicating that the learning ability of epileptic mice was impaired (Figure 4E,F). On the test day (day 6), metrics such as distance in the target quadrant, number of entries in the platform area, percentage of distance traveled in quadrants, time in the platform area, as well as time in the target quadrant were calculated. The 14-28-SC group crossed the platform area less frequently than the control group, indicating significant memory impairment (Figure 4F). Our findings suggest that chronic epileptic mice exhibit significant short-term learning and memory impairment. These results demonstrate that early seizures induce behavioral changes in developmental mice, manifesting as pronounced anxiety, depression, and learning and memory dysfunctions.

### 3.4. Expression and Distribution of GAB_B_R-Relative Microglia After Epilepsy

**Figure 5:**
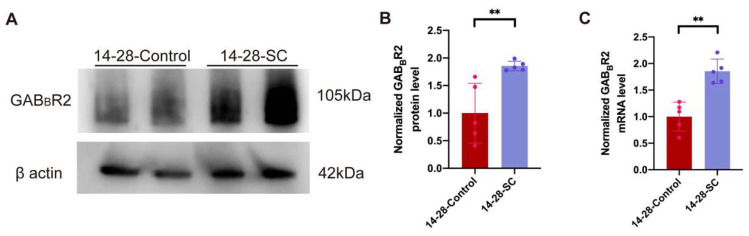
**Expression of GABA_B_2 after epilepsy.** (**A**) Western blot analysis of GABA_B_2 protein level in hippocampal samples from 14-28-Control and 14-28-SC mice. (**B**) Quantifications of GABA_B_2 protein levels. (**C**) The mRNA level of GABA_B_2 in 14-28-Control and 14-28-SC mice were detected by real-time PCR. ** *p* < 0.01; data are reported as the mean  ±  SD; unpaired two-tailed Student’s *t*-test.

To explore the role of GAB_B_R-associated genes in epilepsy, we conducted a WB analysis on hippocampal tissues from control and epilepsy mouse groups aged 14–28 days. The results demonstrated a significant increase in GAB_B_R2 protein expression in the 14-28-SC group (Figure 5A,B). Furthermore, qRT-PCR analysis revealed elevated GAB_B_R2 mRNA levels in 14-28-SC group (Figure 5C).

**Figure 6:**
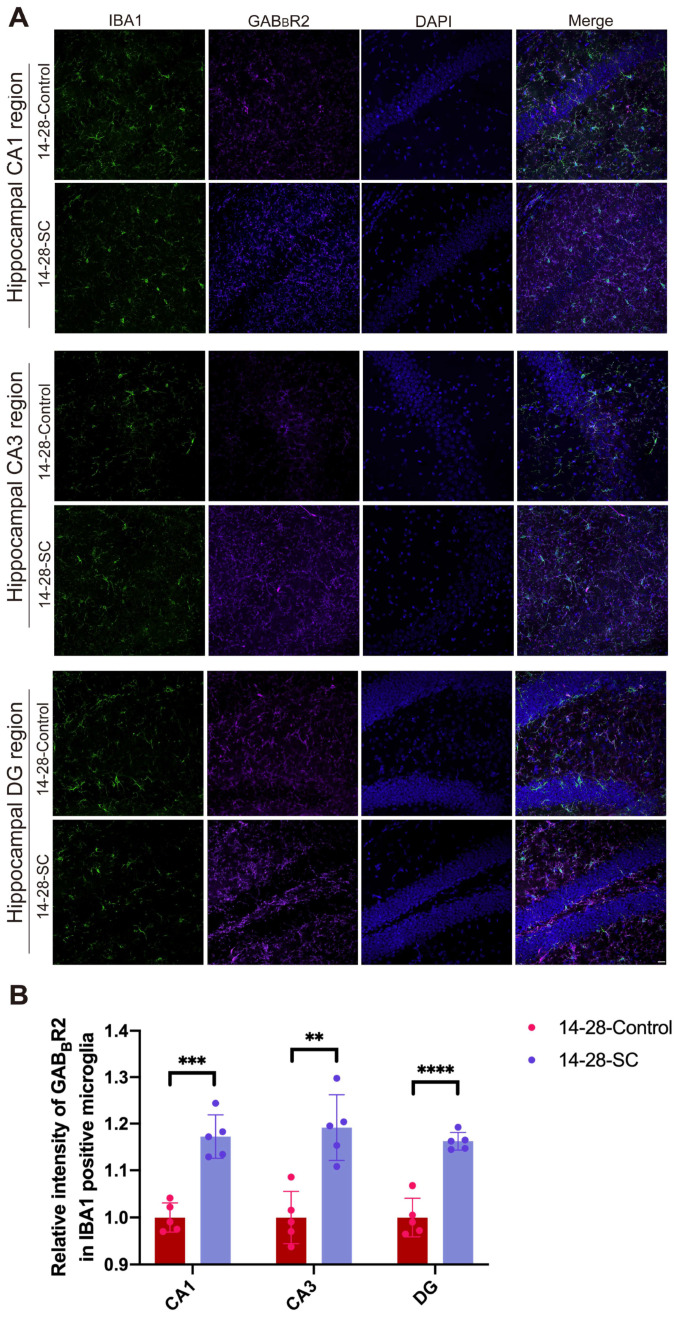
**Distribution and expression of GAB_B_R-relative microglia after epilepsy**. (**A**) Immunofluorescent staining of GAB_B_R2 and IBA1 of hippocampus CA1, CA3 and DG regions in 14-28-Control and 14-28-SC groups. Scale bars: 50 μm. (**B**) Quantifications of the percentage of GAB_B_R2^+^/IBA1^+^ in the hippocampus CA1, CA3 and DG regions. ** *p* < 0.01, *** *p* < 0.001, and **** *p* < 0.0001; data are reported as the mean  ±  SD; unpaired two-tailed Student’s *t*-test.

To investigate the relationship between GAB_B_R-associated microglia and seizures, 14-day-old mice were selected for seizure induction via intraperitoneal injection of KA. Double immunofluorescence staining targeting microglia/GAB_B_R-associated microglia markers IBA1 and GAB_B_R2 was conducted in the hippocampus of both epileptic and control mice 1, 7, 14, and 28 days post injection. The distribution and density of GAB_B_R-associated microglia in the mouse hippocampus were quantified using semi-quantitative immunofluorescence staining. There are no significant differences in the expression of GABAB-receptive microglia within the CA1, CA3, and DG regions of the hippocampus between control and epileptic groups at 1, 7 and 14 days early seizures in 14-day-old mice (Supplemental Figures S2–S4A,B). Results showed that the 14-28-SC group exhibited a significant increase in GAB_B_R-associated microglia within the hippocampal CA1, CA3, and DG regions compared to the 14-28-Control group (Figure 6A,B). These findings suggest that although GAB_B_R-associated microglia do not significantly influence acute seizure events in 14-day-old mice, they are implicated in the progression of chronic epilepsy.

### 3.5. Phagocytic Pruning Effect of GAB_B_R-Receptive Microglia on Neurons After Chronic Epilepsy

**Figure 7:**
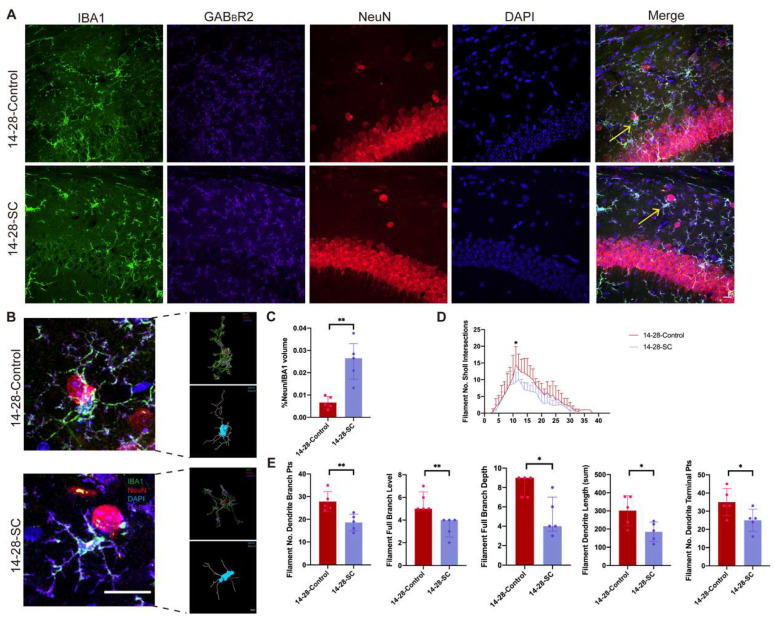
**Phagocytic pruning effect of GAB_B_R-relative microglia on neurons in epilepsy.** (**A**) Immunofluorescent training for GAB_B_R2 (purple), IBA1 (green) and NeuN (red) of hippocampus from 14-28-Control and 14-28-SC group. Arrows indicate 3D reconstructed GAB_B_R-associated microglia. (**B**) Left: Immunofluorescent training for GAB_B_R2, IBA1 and NeuN of hippocampus from 14-28-Control and 14-28-SC group. Scar bar: 30 μm. Right: IBA1/NeuN co-localization in Imaris and IBA1 morphology reconstruction in Imaris. Scar bar: 3 μm. (**C**) Quantifications of IBA1^+^NeuN colocalization using Surface module in Imaris. (**D**) Analysis of GAB_B_R-associated microglial branch using Filament module in Imaris. Statistical analysis was performed using Two-Way ANOVA for (**D**). (**E**) Analysis of total branch, branch point count, terminal point count, branch depth, and branch level in GAB_B_R-associated microglia using Filament module in Imaris. * *p* < 0.05; ** *p* < 0.01; data are reported as the mean  ±  SD; unpaired two-tailed Student’s *t*-test.

Prior research has demonstrated a notable increase in the phagocytic activity of microglia towards neuronal synapses in the KA-induced epilepsy model [43,44]. To further explore the role of GAB_B_R-associated microglia in early epileptic seizures in the developing mouse brain, we used immunofluorescence imaging combined with Imaris software to quantify neuronal content within microglia in the hippocampus of 14-28-Control and 14-28-SC mouse groups. The results revealed that GAB_B_R-associated microglia in the hippocampus of the 14-28-SC group engulfed significantly more neurons compared to the control group (Figure 7A–C), indicating enhanced phagocytic activity. Additionally, morphological analysis showed that the richness, length, and number of terminal points of microglial branches associated with GAB_B_R-associated were reduced in the 14-28-SC group compared to the control group (Figure 7D,E).

These findings suggest that, in the 14-28-SC mouse model induced by intraperitoneal KA injection, GAB_B_R-associated microglia exhibit not only heightened phagocytic activity toward neurons but also increased activation. Collectively, these results implicate GAB_B_R-associated microglia in the phagocytic processes underlying the progression of chronic epilepsy.

## 4. Discussion

This study examined the alterations in the phagocytic function of GAB_B_R-associated microglia in the brains of developing mice with a background of epilepsy, focusing on both acute and chronic epileptic seizures. A key innovation of this research is the application of single-nucleus RNA sequencing (snRNA-seq) to identify cellular subgroup differences in the brains of immature mice with acute and chronic epilepsy, specifically investigating GAB_B_R2 expression within microglial subpopulations. Previous studies have primarily concentrated on the expression and role of GAB_B_R-associated genes in epileptic neurons [45], leaving the functional role of these genes in microglia largely unexplored. This study indicates that, during the chronic phase following epileptic seizures in P14 mice, GAB_B_R2 expression is significantly upregulated in activated microglia within the hippocampus. Notably, we also observed a pronounced increase in the phagocytic function of GAB_B_R-associated microglia in the brains of P14 mice during chronic epileptic seizures. These results highlight the potential importance of GAB_B_R-associated microglia in the phagocytic mechanisms underlying chronic epileptic seizures.

The mechanisms underlying early-onset epilepsy differ significantly from those in mature brains. Childhood epilepsy can disrupt normal brain development and increase the risk of epilepsy later in life [46,47]. Consequently, understanding the pathogenesis of immature epilepsy in rodent models has become a critical area of research. Immature brains exhibit heightened excitability relative to inhibitory processes, resulting in an elevated risk of SE during early developmental stages [47,48]. The P0–P14 period in rodents corresponds to [49,50,51] the neonatal and infant stages of human brain development, making this developmental window ideal for modeling childhood epilepsy [33,48]. Administration of KA via intraperitoneal injection in young rodents (10–14 days old) induces SE and mimics key characteristics of childhood epilepsy in humans [33,52]. SRSs typically manifest by the third week following the induction of an epileptic state [49,50,51]. Our findings corroborate these observations, demonstrating significant SRS in KA-induced epilepsy models, which provide valuable insights into the pathogenesis of epilepsy in immature brains. Furthermore, cognitive and learning deficits have been documented in neonatal epilepsy models, highlighting the severe impact of these comorbidities on patients’ quality of life [50,53,54,55]. It is worth noting that our rodent model of chronic immature epilepsy also exhibits emotional and cognitive impairments, underscoring the importance of neurological comorbidities as a focal point in epilepsy research.

Single-nucleus RNA sequencing (snRNA-seq) technology has significantly advanced the understanding of epilepsy pathogenesis in recent years [23,56]. By employing snRNA-seq to investigate the immune mechanisms underlying epilepsy, researchers have identified a synapse-associated trait formed by activated microglia and T cells in epileptic brain tissue [32]. Notably, due to developmental and site-specific variations, microglia may assume distinct roles in disease progression [57]. In epilepsy, pathological changes in the hippocampus are of particular interest, such as neuronal loss and gliosis [33]. In order to compare the gene expression patterns under steady-state and pathological conditions, we conducted snRNA-seq analysis on hippocampal tissue from mice. Our findings revealed widespread activation of microglia in the hippocampus of immature epilepsy model mice, with microglia transitioning toward a hyperactive, disease-associated phenotype. Previous studies suggest that activated microglia enhance their phagocytic activity following epileptic seizures, potentially mitigating the formation of hyperexcitable neural circuits [58,59]. However, the regulatory factors governing microglial phagocytic function during epilepsy [23,56] remain largely unexplored.

Gamma-aminobutyric acid (GABA) is the primary inhibitory neurotransmitter in the brain, crucial for transmitting inhibitory synaptic signals and maintaining the balance between excitation and inhibition (E/I balance) [60]. Recent studies have shown that GABA receptors, particularly GAB_B_R, influence the functional mechanisms of microglia, facilitating bidirectional communication between neurons and microglia [12,61]. Also, some studies have also found that microglia mainly express GAB_B_R subtype [17,62]. This result is consistent with the analysis of our study. Using hippocampal tissue isolated from 14-day-old mice, we categorized microglia into resting and activated states based on snRNA-seq data. In both types of cells, we observed that GAB_B_R2 expression was markedly higher in activated microglia compared to resting microglia. Subsequently, a comparison of microglia during acute and chronic epilepsy revealed a significant increase in GAB_B_R-expressing microglia during the chronic phase, while no notable changes were observed in the acute phase. The WB and IF experiments further validated this result. These findings are particularly relevant for understanding the role of GABA signaling in the developing brain after epilepsy. Previous studies have shown that GABA enhances the activity of newly formed microglia through intrinsic GAB_B_R signaling [62]. Based on the observed upregulation of GAB_B_R2 in activated microglia, we propose that these cells enhance phagocytosis in the developing brain and may play a pivotal role in the pathophysiology of chronic epilepsy.

To verify this hypothesis, we analyzed morphological changes in GAB_B_R-associated microglia within developing immature brains and their impact on neurons. Our findings reveal that, compared to the control group, GAB_B_R-associated microglia in the epileptic hippocampus during development exhibit a more amoeboid morphology, indicative of increased activation. This suggests an enhancement in the phagocytic function of GAB_B_R-associated microglia in the epilepsy group. Previous studies have shown that microglia engulf dead or damaged neurons or synapses [10,63]. During seizures, neuronal damage may result from excessive excitation or localized metabolic disturbances [64]. Microglia identify and clear these damaged neurons, thereby mitigating further brain injury [65,66]. This process helps protect the brain from further damage. In this study, it was found that there was a significant increase in neurons within GAB_B_R-associated microglia in the epilepsy group, suggesting that GAB_B_R facilitates the phagocytic activity of activated microglia toward neurons during chronic epileptic seizures. Thus, we propose that the heightened neuronal phagocytic activity of GAB_B_R-associated microglia in the hippocampus may contribute significantly to the progression of immature brain disorders in the context of epilepsy.

In summary, our study emphasizes the phagocytic role of GAB_B_R-associated microglia in the progression of developmental epilepsy. However, several limitations should be acknowledged. First, while we elucidated the phagocytic function of GAB_B_R-associated microglia in immature brains, it remains unclear whether these microglia specifically target excitatory or inhibitory neurons. Second, previous studies on GAB_B_R-associated microglia have shown their involvement in the remodeling of inhibitory synaptic connections [67]. Further research is required to elucidate the precise mechanisms through which GAB_B_R-associated microglia contribute to epilepsy. Notably, the detailed snRNA-seq analysis conducted in this study provides valuable insights into the critical role of microglia in developmental epilepsy and offers new perspectives for the prevention and treatment of this condition.

## 5. Conclusions

GAB_B_R-associated microglia significantly contribute to the progression of immature brain diseases by promoting neuronal phagocytic activity.

## Data Availability

The datasets used and/or analyzed during the current study are available from the corresponding author on reasonable request. The raw data supporting the conclusions of this article will be made available by the authors, without undue reservation.

## Supplementary Materials

The following supporting information can be downloaded at: https://www.mdpi.com/article/10.3390/biomedicines13020269/s1, Figure S1: Original Western blotting images of Figure 5; Figure S2: Distribution and expression of GABBR2 between 14-1-Control and 14-1-SC; Figure S3: Distribution and expression of GABBR2 between 14-7-Control and 14-7-SC; Figure S4: Distribution and expression of GABBR2 between 14-14-Control and 14-14-SC.
