# Supplementary material for: Phagocytic Function Analyses of GABBR-Related Microglia in Immature Developing Epileptic Brain Based on 10× Single-Nucleus RNA Sequencing Technology
Source: Biomedicines. 2025 Jan 22;13(2):269. doi: 10.3390/biomedicines13020269 (PMC11853619; doi:10.3390/biomedicines13020269)

Supplement Figure S1. Original Western blotting images of Figure 5.

GAB_BR2

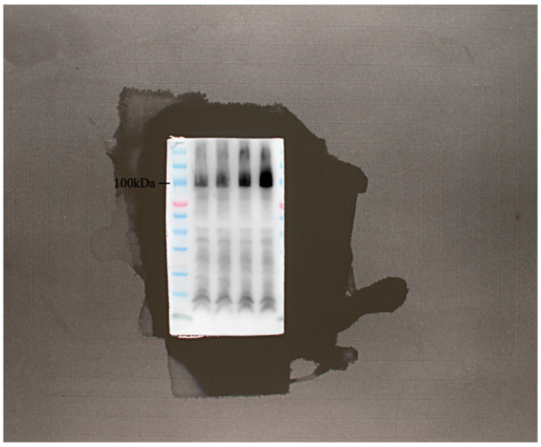

β actin

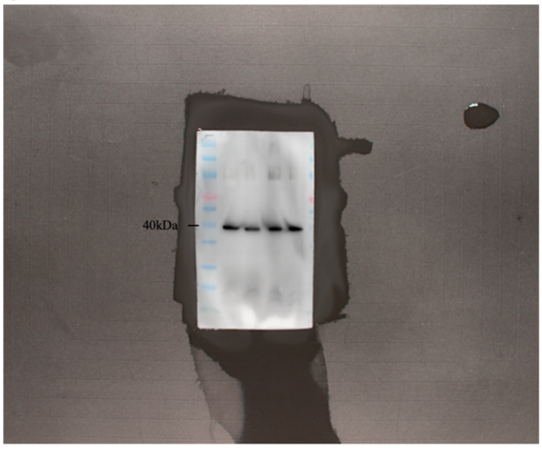

GAB_BR2

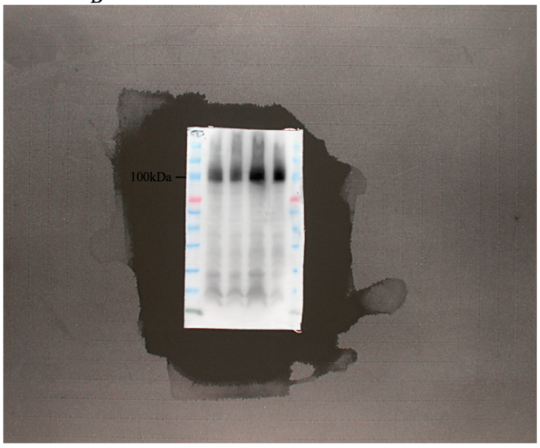

β actin

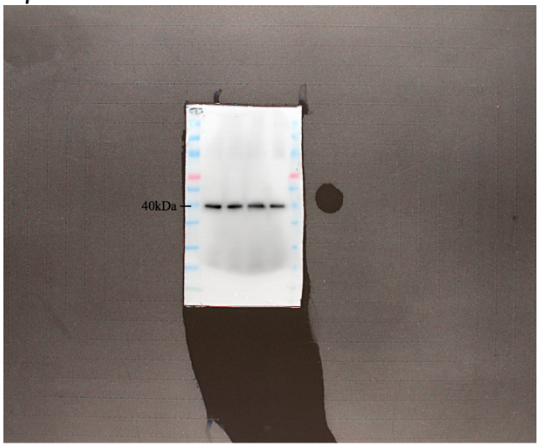

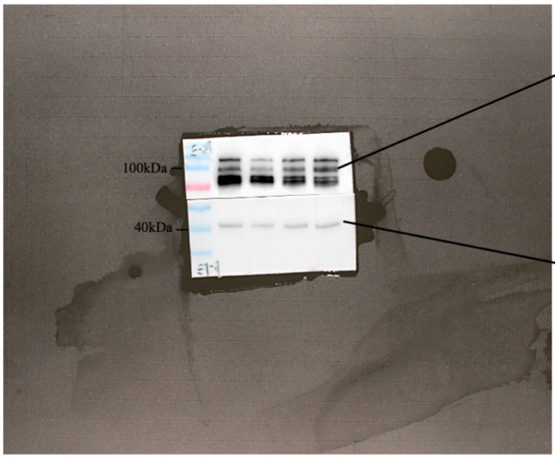

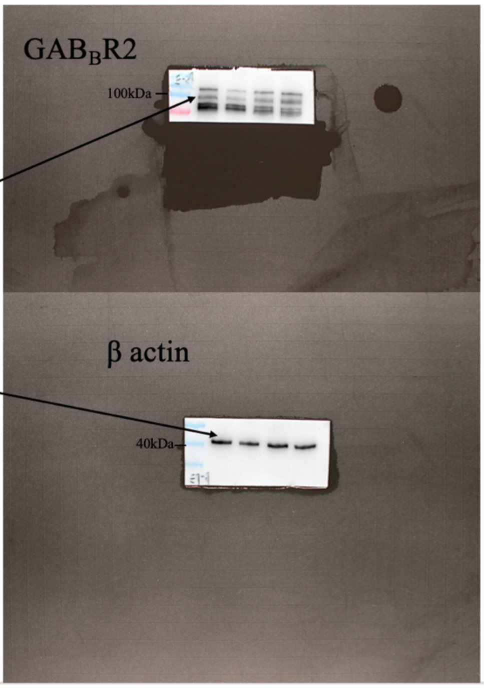

Supplement Figure S2. Distribution and expression of GAB$_B$R2 between 14-1-Control and 14-1-SC.

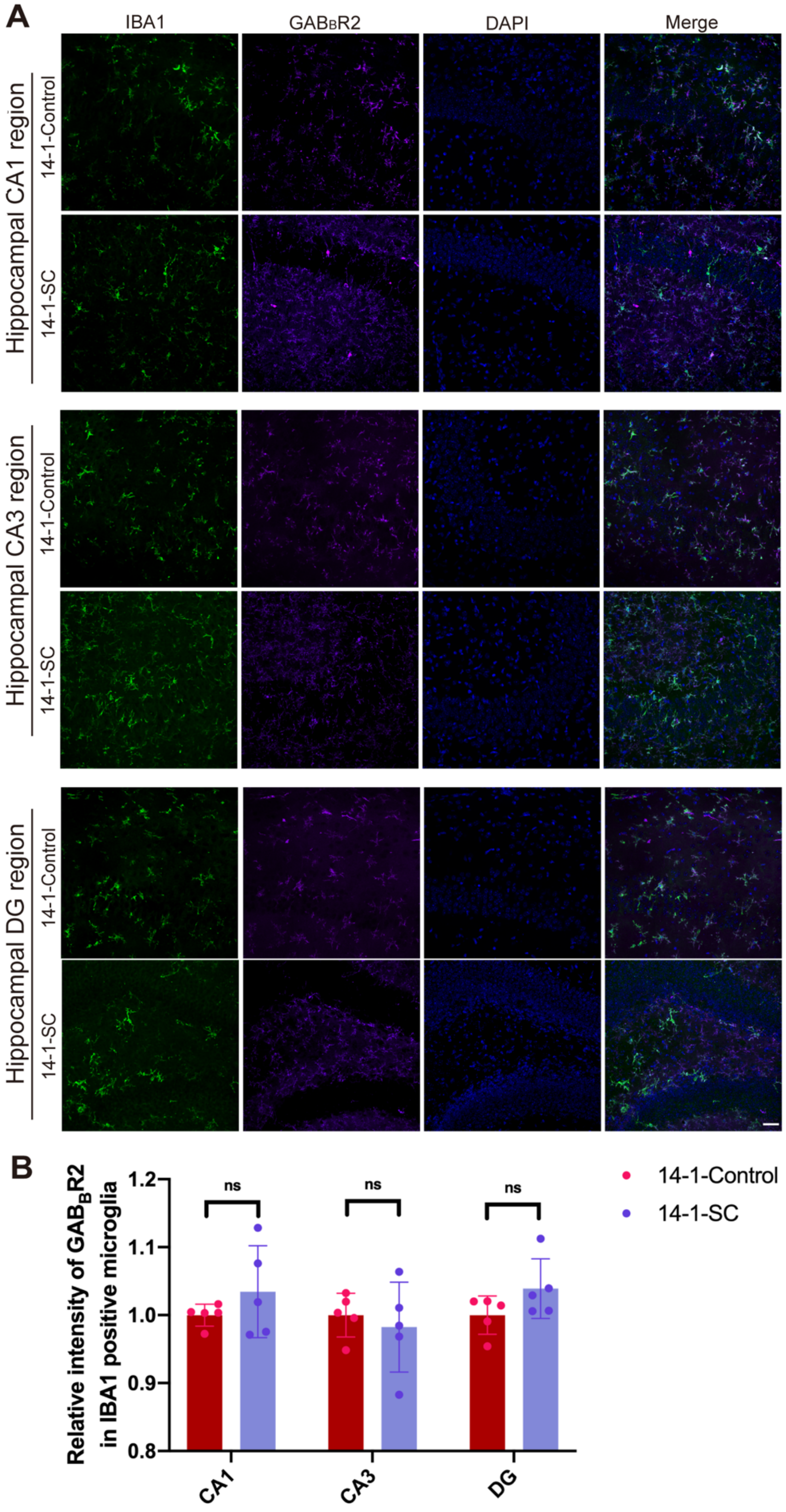

Supplement Figure S3. Distribution and expression of GAB$_B$R2 between 14-7-Control and 14-7-SC.

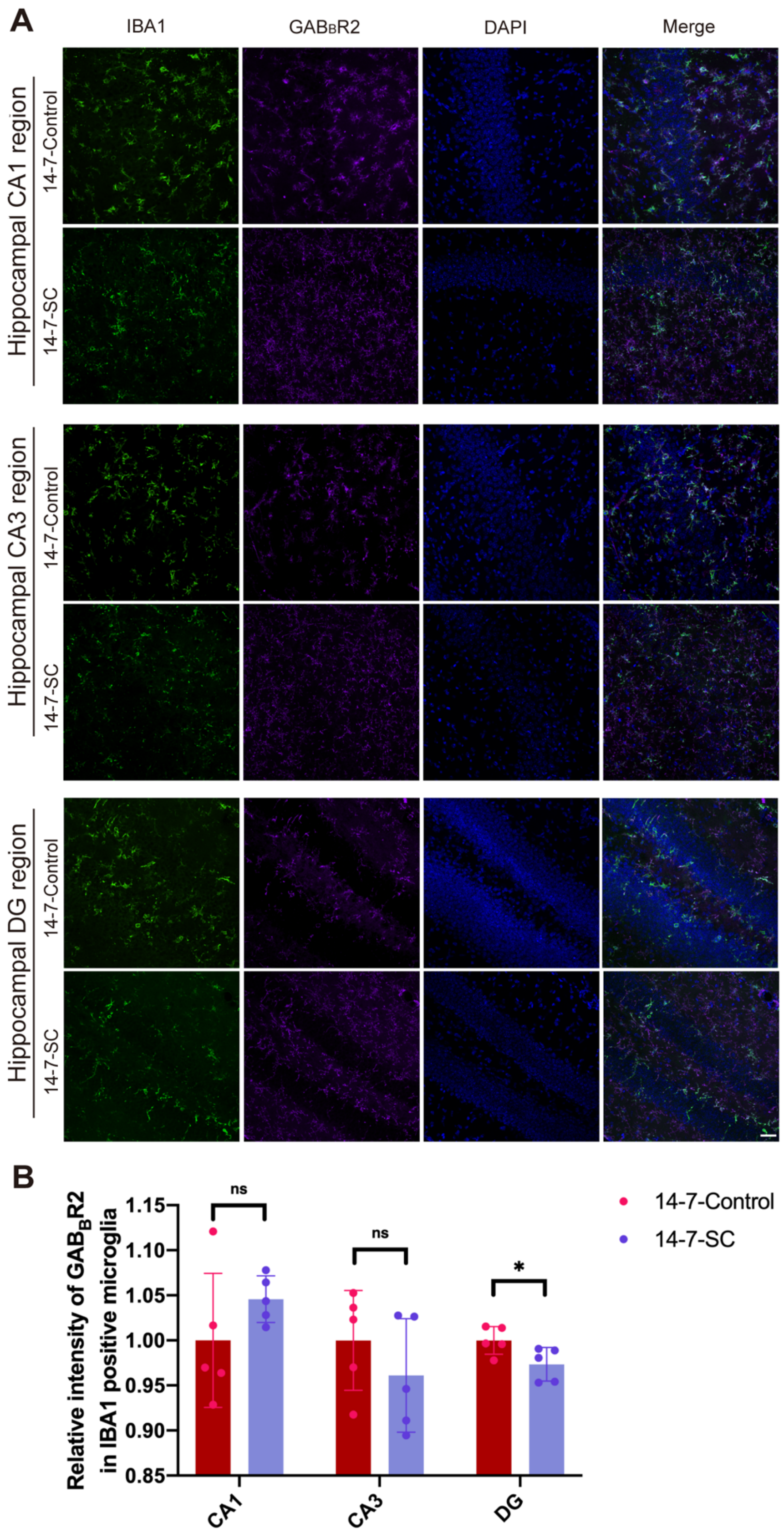

Supplement Figure S4. Distribution and expression of GAB$_B$R2 between 14-14-Control and 14-14-SC.

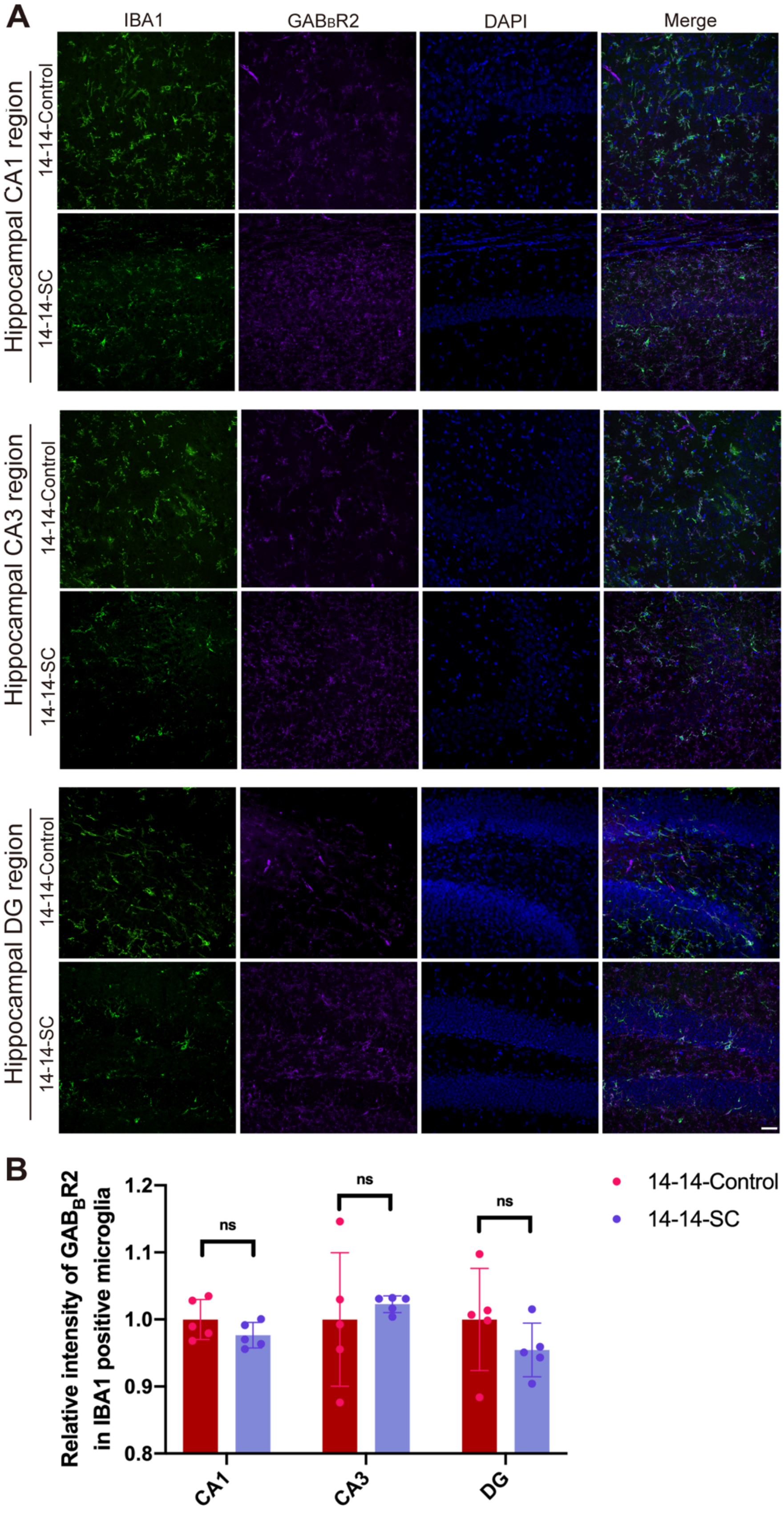

Supplement: Supplementary file 1 [file biomedicines-13-00269-s001.zip › biomedicines-3437267-supplementary.pdf]
